# Supplementary material for: The Prevalence of MRI-Defined Sacroiliitis and Classification of Spondyloarthritis in Patients with Acute Anterior Uveitis: A Longitudinal Single-Centre Cohort Study
Source: Diagnostics (Basel). 2022 Jan 11;12(1):161. doi: 10.3390/diagnostics12010161 (PMC8774303; doi:10.3390/diagnostics12010161)
Supplement: Supplementary file 1 [file diagnostics-12-00161-s001.zip › diagnostics-1509332-supplementary.pdf]

diagnostics

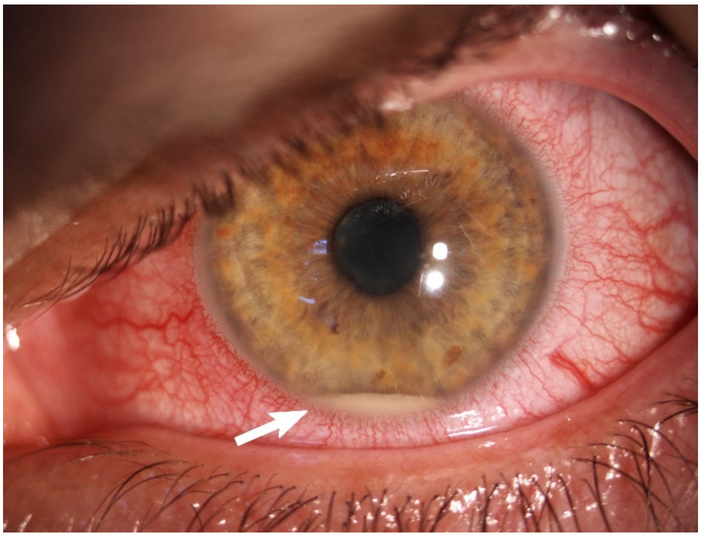

Supplementary Figure S1. Acute anterior uveitis of the left eye. Mixed conjunctival and ciliary injection, sedimentation of inflammatory material in the anterior chamber—hypopyon (arrow).

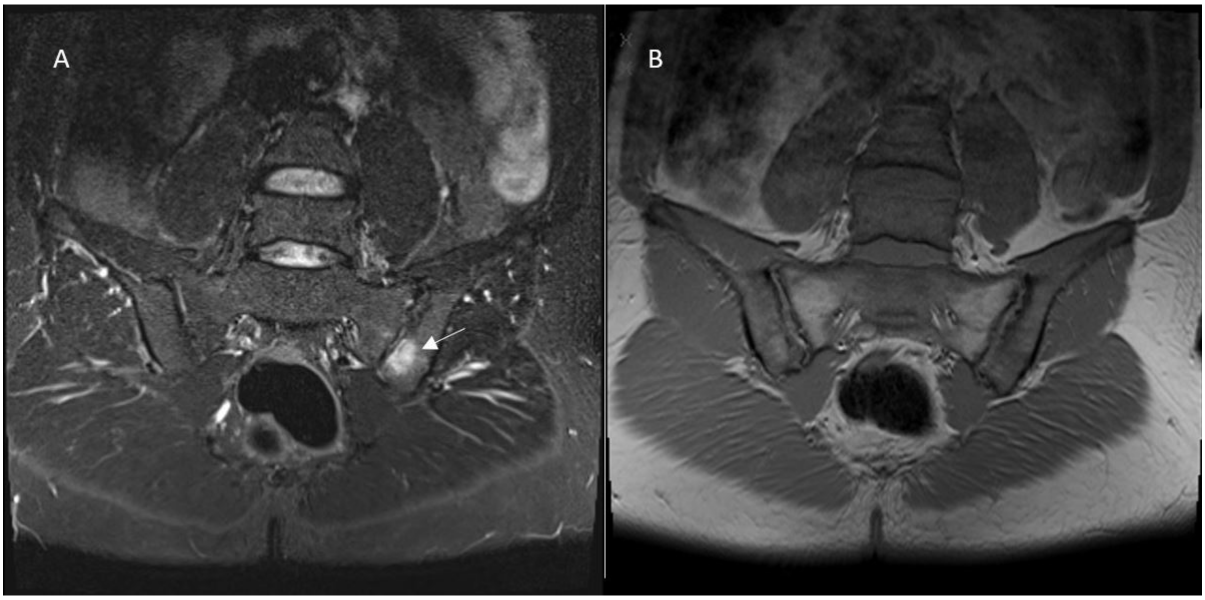

Supplementary Figure S2. (**A**). STIR: Bone marrow edema on the iliac part of left sacroiliac joint which is highly suggestive of axSpA. (**B**). Corresponding T1W image.
